# Exploring Barriers and Facilitators to the Implementation of Nurse-Driven Catheter-Associated Urinary Tract Infection Prevention Protocols in Intensive Care Units in Saudi Arabia: A Qualitative Study

**DOI:** 10.3390/healthcare14121741

**Published:** 2026-06-17

**Authors:** Nader E. Alotaibi, Ahmed S. Alsadoun

**Affiliations:** Department of Medical Surgical of Nursing, College of Nursing, King Saud University, Riyadh 12372, Saudi Arabia

**Keywords:** catheter-associated urinary tract infection (CAUTI), nurse-driven protocols, intensive care units, infection prevention and control, implementation barriers and facilitators, implementation science, CFIR, qualitative research, Saudi Arabia, patient safety

## Abstract

Background: Catheter-associated urinary tract infection (CAUTI) remains a major healthcare-associated infection, particularly in intensive care units (ICUs). Nurse-driven urinary catheter removal protocols can reduce catheter duration and improve patient safety; however, their implementation remains inconsistent. This study explored barriers and facilitators influencing the implementation of nurse-driven CAUTI prevention protocols in Saudi ICUs. Methods: A qualitative study guided by the Consolidated Framework for Implementation Research was conducted in two tertiary hospitals in Riyadh, Saudi Arabia. A purposive sample of 23 ICU nurses, infection control nurses, and nurse managers participated in semi-structured interviews. Data were analyzed using Braun and Clarke’s thematic analysis approach, supported by NVivo. Results: Two overarching themes emerged. Facilitators included interprofessional communication and shared decision-making, clinical experience, and professional commitment. Barriers included workload and staffing pressures, physician dominance and nurse hesitation, knowledge deficits and poor adherence to guidelines, inconsistent documentation, and reduced awareness of catheter presence. Implementation was influenced by interconnected individual, organizational, and cognitive factors. Conclusions: Implementation of nurse-driven CAUTI prevention protocols is shaped by both enabling and limiting factors. Strengthening interprofessional collaboration, supporting nurse autonomy, improving documentation, and providing ongoing education may enhance protocol uptake and sustainability, ultimately improving patient safety.

## 1. Background

Urinary tract infection (UTI) is one of the most prevalent infections occurring in healthcare settings, which are mostly linked to urinary catheter use [1]. Catheter-associated urinary tract infection (CAUTI) remains one of the most common healthcare-associated infections (HAIs) [2,3].

Definitions of CAUTI differ internationally. The Infectious Diseases Society of America (IDSA) defines catheter-associated urinary tract infection (CAUTI) as a condition in which a patient presents with clinical signs or symptoms consistent with a urinary tract infection, without an alternative identified source of infection, accompanied by microbiological evidence of significant bacteriuria, defined as the growth of ≥10^3^ CFU/mL of at least one bacterial species in a single urine specimen obtained from a catheter sample or from a midstream voided urine specimen collected within 48 h after removal of a urethral, suprapubic, or condom catheter [4].

However, a widely accepted definition describes CAUTI as a urinary tract infection occurring in a patient who has had an indwelling urinary catheter in place for more than two days, along with at least one clear clinical sign of infection [5]. These signs may include fever (above 38 °C), suprapubic tenderness, or pain at the costovertebral angle, in addition to a urine culture showing no more than two types of microorganisms [3,5]. It is estimated that about 12% to 16% of hospitalized adults receive an indwelling urinary catheter (IUC), often referred to as a Foley catheter, during their hospital stay, and the risk of infection rises with each additional day the catheter stays in place [2,6,7]. These infections are linked to longer hospitalizations and increased healthcare costs. Because of this burden, CAUTI prevention has become a major priority for the Centers for Medicare and Medicaid Services (CMS) through its Hospital-Acquired Condition Reduction Program, which reduces reimbursement for hospitals performing poorly on HAI measures, including CAUTI rates [5,7,8].

Evidence-based guidelines offer clear and practical direction for healthcare professionals on how to use, monitor, and remove indwelling urinary catheters (IUCs) safely and appropriately [9,10,11]. Nevertheless, translating these recommendations into routine practice remains challenging, and the prevention of CAUTIs continues to be difficult to achieve consistently [12].

To overcome this gap, nurse-driven protocols for catheter removal have been adopted as a clinical solution. These protocols assist nurses with removing catheters independently when particular clinical criteria are met, which reduces reliance on direct physician involvement [2,13]. Emerging evidence indicates that these protocols help reduce the duration of catheter use and improve the quality of patient care [14,15].

Despite these advantages, the uptake of nurse-driven urinary catheter removal protocols in acute care settings remains uneven [2,16]. A range of factors contribute to this variability, including patient and family expectations to maintain catheterization, cultural norms that support prolonged use, the convenience of leaving catheters in place in busy clinical settings, and nurses’ uncertainty or lack of confidence in applying these protocols autonomously [2,17].

A better understanding of how hospitals implement nurse-driven urinary catheter removal protocol is important for improving healthcare-associated infection (HAI) prevention. It can also help identify implementation gaps, clarify barriers to effective protocol use, and guide efforts to reduce CAUTI rates [14,18,19].

Therefore, this qualitative study offers a context-specific understanding of the barriers and facilitators influencing the implementation of nurse-driven CAUTI prevention protocols in intensive care units (ICUs) in Saudi Arabia. The findings can be utilized to implement locally tailored interventions and ICU policies, optimize nursing practice, and guide targeted training initiatives, which ultimately improves patient safety, reduce healthcare expenditure, and promote the quality of care.

## 2. Methods

A qualitative study design was used to explore barriers and facilitators that affect the implementation of nurse-driven CAUTI prevention protocols. The study was guided by the Consolidated Framework for Implementation Research (CFIR) [20]. This approach was adopted to highlight the intricate interplay between clinical experiences and organizational contexts within ICU settings. Additionally, this method was used to explore nurses’ clinical experiences in ICUs in Saudi Arabia, providing a deeper understanding of the organizational and professional contexts that have an influence on healthcare quality.

### 2.1. Setting

This study was performed in adult ICUs in two tertiary hospitals in Riyadh, Saudi Arabia. These units care for critically ill patients and are recognized as high-risk environments for CAUTIs. This makes them an important setting for exploring how nurse-driven catheter removal protocols are applied and what shapes consistency in practice and patient safety.

### 2.2. Participants and Sampling Strategy

Participants included were registered nurses who work in ICUs and are directly engaged in patient care or infection prevention practices. A purposive sampling strategy was used to obtain maximum variation and reflect diverse perspectives across professional roles, clinical experience, and educational backgrounds. Eligible participants comprised nurses working in ICU and infection control departments, and nurse managers who were involved in catheter care or in supporting CAUTI prevention implementation. Inclusion criteria required at least two years of ICU experience and the capability to share informed, experience-based insights into CAUTI prevention practices. Recruitment was conducted in parallel with data analysis until thematic saturation was attained, at which point no additional interviews produced new themes or meaningful insights.

### 2.3. Data Collection Procedures

Semi-structured, in-depth interviews were employed as the primary data collection method. Interviews were conducted by the principal investigator, who is trained in qualitative research methods, with support from a research team member responsible for logistical and technical aspects. Prior to data collection, participants were provided with information about the study purpose, procedures, and confidentiality measures, and written informed consent was obtained. An interview guide was used to explore experiences with urinary catheter management, implementation of CAUTI prevention protocols, and organizational influences, including training, accountability, and workload. The interview guide consisted of open-ended questions designed to explore participants’ experiences with nurse-driven urinary catheter protocols, decision-making processes, and organizational influences on implementation. The questions were structured to capture key implementation domains, including individual factors (e.g., knowledge, confidence, and professional role), interpersonal dynamics (e.g., communication and collaboration with physicians), and organizational context (e.g., institutional support, workload, and policies).

Example questions included: “Can you describe your experience with nurse-driven urinary catheter protocols in your ICU?”, “How do you decide when a urinary catheter should be removed under this protocol?”, “What factors make it easier to apply the nurse-driven protocol?”, “What challenges or barriers have you encountered when implementing the protocol?”, “How do physicians and other team members respond to nurse-led catheter removal decisions?”, and “How does institutional support (e.g., policies, leadership, and training) influence your ability to follow the protocol?” Additional probing questions were used as needed to clarify responses and deepen understanding, such as “Can you tell me more about that?” and “Can you provide an example?”

Interviews were conducted in a private setting within the ICU to ensure confidentiality and participant comfort. Each interview lasted between 30 and 50 min and was audio-recorded with participants’ permission. Data collection proceeded until thematic saturation was achieved. Thematic saturation was assessed alongside data collection and analysis. Interviews were transcribed and coded soon after completion, with new codes continuously compared to earlier ones. Saturation was considered achieved when no new themes emerged across successive interviews. This was documented in analytic notes and confirmed through regular team discussions, indicating that additional interviews were unlikely to provide new insights.

The interview guide was developed by the research team following a careful review of the relevant literature on catheter-associated urinary tract infection (CAUTI) prevention and implementation science. It was further shaped by key domains of the Consolidated Framework for Implementation Research (CFIR), which helped ensure that important implementation-related factors were adequately explored. The guide aimed to capture participants’ experiences with urinary catheter management, as well as their perspectives on the barriers and facilitators influencing the implementation of nurse-driven protocols, and the broader organizational and contextual factors affecting practice. To enhance its clarity and relevance, the interview guide was reviewed by experts in qualitative research.

### 2.4. Data Analysis

Data were analyzed using Braun and Clarke’s thematic analysis approach [21]. The analysis was guided by an interpretivist perspective, which assumes that meaning emerges through the interaction between participants’ experiences and the researchers’ interpretation of the data. The Consolidated Framework for Implementation Research (CFIR) was used as a guiding lens to help interpret factors related to implementation, while thematic analysis allowed us to identify recurring patterns and meanings across the interview data.

The analysis was carried out in several steps. First, the researchers immersed themselves in the data by repeatedly listening to the audio recordings and reading the transcripts to become closely familiar with participants’ accounts. Next, one author conducted line-by-line coding of all transcripts in NVivo, identifying meaningful segments of text that reflected barriers, facilitators, and contextual influences on protocol implementation. These initial codes were then compared and grouped into candidate themes and subthemes based on shared meaning and relevance across the data. The themes were then reviewed and refined by checking them against the coded extracts and the full dataset to ensure they were coherent and distinct. After that, the themes were clearly defined and named to capture the main ideas they represented. Finally, the themes were brought together and presented in Section 3 (Results), supported by representative quotations from participants. In the later stages, CFIR was used to help interpret and organize the findings within a clear framework. This approach ensured that the analysis stayed true to participants’ perspectives while also being supported by a well-established theoretical model. To strengthen the rigor of the analysis, transcript review, coding, and theme development were discussed within the research team, and any differences in interpretation were resolved through discussion until agreement was reached.

### 2.5. Rigor

A range of measures were implemented to enhance the rigor of the study and to minimize the potential influence of researcher bias across data collection, analysis, and reporting. Credibility was supported by establishing rapport with participants during the interviews, allowing sufficient time to gain a meaningful and contextually grounded understanding of their experiences within the ICU setting. The research team also maintained close engagement with the data through repeated reading of the transcripts and careful listening to the audio recordings, which facilitated a deeper and more nuanced interpretation of participants’ accounts.

To strengthen dependability and confirmability, interview recordings were reviewed multiple times and systematically compared with the verbatim transcripts and the developing themes. This iterative process ensured that the analysis remained firmly anchored in participants’ perspectives rather than being shaped by prior assumptions. Furthermore, the involvement of multiple researchers in reviewing transcripts, coding, and theme development enabled ongoing cross-checking, thereby reducing the risk of individual bias and enhancing the consistency of the findings. These strategies contributed to maintaining methodological rigor and ensuring credibility, dependability, and overall trustworthiness of the study.

### 2.6. Ethical Considerations

Ethical approval was granted by the King Saud University Institutional Review Board (approval number: KSU-HE-26-0170). Formal permissions were also obtained from the participating hospitals before the commencement of data collection. After receiving the necessary permissions, eligible ICU nurses were approached and invited to participate in the study. They were provided with a detailed information sheet outlining the study objectives, procedures, and their expected involvement. Written informed consent was obtained from all participants before data collection, and consent for audio recording was also secured. Participants were clearly informed that their participation was entirely voluntary and that they had the right to withdraw at any stage without any form of penalty or impact on their professional duties.

Strict measures were taken to ensure confidentiality and privacy. No identifying information, such as participant names, was collected. Instead, each participant was assigned a unique code to maintain anonymity. All electronic data were securely stored on password-protected devices accessible only to the research team. Furthermore, interviews were conducted in private, quiet locations within the ICU setting to ensure discretion and participant comfort. These procedures were implemented to protect participants’ rights and maintain the ethical rigor of the study. A completed COREQ checklist is provided as Supplementary File S1 to enhance transparency and support comprehensive reporting of the qualitative methods and findings.

## 3. Results

### 3.1. Participant Demographic and Professional Characteristics

The study included 23 participants, primarily ICU nurses (69.6%), along with infection control nurses (17.4%) and nurse managers (13.0%). Most participants were female (65.2%). Nearly half were aged 30–39 years (47.8%), while 30.4% were younger than 30 years and 21.7% were aged 40 years or older. Participants had varied ICU experience, with 39.1% reporting 6–10 years, 34.8% reporting 11–20 years, and 26.1% reporting 2–5 years of experience. All participants were working in ICU settings across the two study hospitals (Table 1).

### 3.2. Thematic Findings

Thematic analysis generated two overarching themes. The first theme, facilitators of CAUTI prevention implementation, comprised three subthemes: interprofessional communication and shared decision-making, clinical experience, and professional commitment and responsibility. The second theme, barriers to CAUTI prevention implementation, comprised five subthemes: workload and staffing pressures, physician dominance and nurse hesitation, knowledge deficits and suboptimal adherence to guidelines, inconsistent documentation and lack of standardization, and cognitive overload with reduced awareness of catheter presence (Table 2). Collectively, these themes illustrate the multifaceted and interrelated factors that influence urinary catheter management practices within ICU settings in Saudi Arabia.

**Theme 1: Facilitators of CAUTI prevention implementation**

**Subtheme 1.1: Interprofessional communication and shared decision-making**

Participants described that good communication and teamwork contributed to enhanced adherence to catheter infection prevention, and their joint discussions helped the team reach decisions quickly and together. Open communication with physicians also assisted with increasing nurses’ confidence in advocating for catheter removal, particularly when the plan was well defined. Infection control teams played a significant role in highlighting the importance of prevention and making catheter assessment a priority.

*“During clinical rounds, we all discuss the patient’s condition, making it clear to everyone if catheterization is necessary. The decision is made collectively, not individually.”*

(P3)

*“When communication with the physician is open, I feel more confident requesting catheter removal, which makes the process easier and faster.”*

(P11)

*“Clear communication fosters confident decision-making, especially when the plan is understood by everyone.”*

(P7)

*“Involving the infection control team elevates the importance of catheter evaluation, making it a priority rather than just a routine task.”*

(P15)

**Subtheme 1.2: Adequate experience of staff**

Participants reported that the accumulation of clinical experience helped increase nurses’ self-confidence and independence, while also enhancing their clinical intuition in catheter use. With experience, their dependence on physicians decreased, and they became more decisive and less hesitant, reflecting a trend toward greater professional independence. Furthermore, more experienced nurses exhibited enhanced competence and confidence in reassessment, grounded in a thorough understanding of risk, which supported prompt, evidence-based decision-making.

*“With time and experience, the nurse knows when a catheter can be removed easily and confidently.”*

(P6)

*“Initially, I relied on the doctor, but experience has made me more confident in making the decision independently.”*

(P14)

*“Experience provides greater confidence in the assessment, so the catheter is not removed based on mere suspicion.”*

(P2)

*“Experienced nurses often make decisions more quickly because they have a better understanding of the risks.”*

(P9)

**Subtheme 1.3: Professional commitment and responsibility**

The nurses expressed that being responsible and committed to maintaining patients’ safety supported their adherence to catheter-associated infection prevention measures. They also reported that regular catheter assessment is part of their ethical responsibility toward patients, and this helped in taking initiative and acting consistently, rather than just following instructions. Participants stated that past experiences with potential health complications assisted them with strengthening their sense of responsibility and accountability, which contributed to careful management of catheters without delay.

*“I always check whether the patient really needs the catheter. It’s my responsibility to monitor it and remove it when it’s no longer necessary.”*

(P5)

*“My job is not just about following orders; it is also my professional duty to maintain patient safety and prevent infections.”*

(P12)

*“Even if there are no reminders, verifying the necessity of the catheter is a critical issue and it’s my professional role because we are working in the ICU to provide the best care for the patient.”*

(P1)

*“From my previous experiences with potential health problems associated with catheters, I consider catheterization as a serious issue and treat it with caution; I also remove it immediately when it’s no longer required.”*

(P17)

**Theme 2: Barriers to CAUTI prevention implementation**

**Subtheme 2.1: Heavy workload and staffing pressures**

Participants expressed that heavy workloads and staff shortages in the ICU reduce nurses’ focus on catheter reassessment. They also reported that dealing with patients who are critically ill and unstable can lead to delayed or missed catheter reassessment.

*“When we are extremely busy, especially with critically ill patients, catheter reassessment may not be prioritized.”*

(P8)

*“Staff shortages increase the difficulty of following guidelines completely, even if we are willing to do so.”*

(P4)

*“In emergency situations, we mainly concentrate on saving the patient’s life, so checks are often deferred.”*

(P13)

*“Work pressure and numerous tasks in the ICU make it difficult for us to monitor the catheter condition regularly.”*

(P10)

**Subtheme 2.2: Physician dominance and nurse hesitation**

Nurses reported reluctance to remove urinary catheters independently, as physician approval was perceived as necessary prior to action. Even when institutional protocols permitted nurse-led removal, participants described delaying decisions until confirmation was obtained from physicians to avoid potential conflict. This reliance on physician authorization often resulted in unnecessary prolongation of catheterization without clear clinical indication.

*“If the doctors want to keep the catheter inserted for longer, we often follow their decision to avoid any arguments or conflict.”*

(P16)

*“Even if we are allowed to remove the catheter, in our culture physician’s decision comes first, so nurses hesitate to act independently.”*

(P6)

**Subtheme 2.3: Knowledge deficits and poor adherence to guidelines**

Participants stated that insufficient knowledge and limited understanding of the guidelines targeting urinary catheter infection prevention have negative effects on their adherence. They also noted that uncertainty regarding appropriate use of the catheter can contribute to keeping the catheter in longer than indicated, while limited knowledge leads to suboptimal implementation of protocol. Additionally, poor training causes nurses to depend on old practices rather than evidence-based approaches, which may delay timely decision-making.

*“The problem is that not everyone is familiar with the protocol, so the catheter remains in place for a long time without considering potential complications.”*

(P18)

*“Some nurses do not follow procedures completely, possibly due to a lack of knowledge, and they need more training to provide optimal care for patients with urinary catheters.”*

(P2)

**Subtheme 2.4: Inconsistent documentation and lack of standardization**

Participants indicated that inconsistent documentation with unclear information prevented effective management of the urinary catheter. Furthermore, poor documentation and inconsistent patient information contributed to ambiguity and uncertainty in catheter removal decisions and increased confusion among nurses.

*“Sometimes a catheter is present, but the reason isn’t clearly documented, leaving us unsure of how to proceed.”*

(P19)

*“Different documentation methods among nurses create confusion.”*

(P7)

*“During the shift handover, if the situation is not clear, we might assume the catheter should remain.”*

(P20)

*“Standardized documentation facilitates understanding of correct procedures and supports decision-making.”*

(P15)

**Subtheme 2.5: Cognitive factors and lack of awareness of catheter presence**

Nurses described that the intense workload, cognitive overload, psychological distress, and the challenges of caring for seriously ill patients in the high-pressure unit can contribute to missed catheter reassessments. Additionally, important routine tasks can be missed inadvertently, which can compromise their adherence to urinary catheter infection prevention measures.

*“When we are very busy, under pressure, and have many tasks to complete during the shift, we forget to pay attention to the urinary catheter.”*

(P21)

*“Because of the complex and critical nature of illnesses in intensive care, we often view urinary catheterization as a simple procedure and may underestimate its serious complications.”*

(P9)

## 4. Discussion

This study provides an in-depth and contextualized understanding of the barriers and facilitators that influence the implementation of nurse-driven protocols for the prevention of CAUTI in ICUs in Saudi Arabia. The findings were interpreted using the CFIR framework to better understand the multiple factors influencing CAUTI prevention. Themes related to nurses’ experience and responsibility align with individual characteristics, while communication and teamwork reflect the inner setting. Workload and documentation relate to organizational factors, and physician influence reflects implementation processes. Using CFIR in this way helps clarify the findings and highlights their relevance for improving infection prevention practices. The findings indicate that implementation is shaped by interconnected factors, including knowledge and awareness, interprofessional communication, autonomy, workload, documentation, and cognitive attention to catheter presence. This pattern is in line with previous studies showing that these factors are interrelated rather than isolated, and have a significant impact on capability, opportunity, and motivation in clinical practice [7,22].

### 4.1. Facilitators of CAUTI Prevention Implementation

This study showed that interprofessional communication and shared decision-making were facilitating factors in implementation. These findings reflect the influence of the inner setting, particularly team communication and organizational culture, in shaping implementation practices. Nurses demonstrated greater confidence in advocating for catheter removal when engaged in collaborative team discussions. This finding is supported by DePuccio, Gaughan [2] who found that successful implementation of nurse-driven urinary catheter protocols was determined by several factors such as regular communication, shared understanding of removal authority, and staff buy-in. Another study found that catheter removal was deferred in the absence of active discussion regarding catheter status during clinical rounds [23]. This finding highlights the importance of integrating catheter reassessment into routine rounds instead of being considered a non-mandatory component of care.

The present study also identified the adequate nursing experience, professional commitment, and responsibility as enabling factors. This highlights the role of individual-level factors, including knowledge, experience, and confidence, in influencing adherence to evidence-based practices. Nurses with greater experience were more confident and independent in reassessing the necessity for catheters or undertaking any action, which indicated improved clinical judgment and increased professional self-confidence. This finding is congruent with evidence-based implementation guidance from the Centers for Disease Control and Prevention and the Agency for Healthcare Research and Quality [24,25,26], which emphasize the role of nurses in reassessment of catheter necessity and timely removal of unnecessary catheters. The Agency for Healthcare Research and Quality highlights nurse-led catheter removal as a practical and safe approach, and the Centers for Disease Control and Prevention emphasize removing catheters as soon as they are no longer needed.

### 4.2. Barriers to CAUTI Prevention Implementation

Our study found various barriers, including heavy workload, staffing pressures, and cognitive overload. Participants expressed that urgent clinical demands within the ICU often took priority over catheter reassessment, and the complexity of clinical workloads increased the likelihood of missed or delayed routine reviews. These findings reflect broader organizational influences that may limit the consistent application of prevention protocols in high-acuity settings. From a CFIR perspective, this corresponds to inner setting factors, especially structural characteristics and implementation climate, where heavy workload, staffing limitations, and competing demands in clinical practice affect catheter reassessment practices in ICU settings.

These findings are supported by previous studies reporting that heavy workload and limited staffing contribute to reduced adherence to CAUTI prevention practices [27,28,29]. Studies conducted by Fasugba, McInnes [30] and Wu, Bai [22] found that workload pressures, inadequate prioritization, and restricted opportunities for catheter reassessment are major barriers to UTI prevention and early catheter removal. Additionally, another study also reported that catheter removal is frequently given lower priority in high-demand clinical settings, particularly when it is unclear whether a catheter is in place or documentation is insufficient [23]. Staffing constraints also reduce access to training, undermining nurses’ knowledge and use of current practice guidelines [31]. Engaging healthcare workers in CAUTI prevention remains challenging, partly because these infections are often seen as less critical than others and because reducing catheter use is perceived to increase workload—findings consistent with earlier studies [23,32,33]. Our results also show the complexity of balancing and prioritizing safety practices, often influenced by varying perceptions. These findings indicate that disengagement extends beyond convenience; it reflects broader concerns that require targeted attention and understanding [23].

Our findings identified physician dominance and nurse hesitation as major barriers to implementation. Nurses indicated their reluctance to remove catheters without obtaining approval from physicians, despite the availability of protocols that enable nurse-driven intervention. This finding is consistent with a study of DePuccio, Gaughan [2], who identified nurses’ deference to physicians and resistance from physicians as significant barriers, hindering the implementation of nurse-led protocols. Another study reported that successful implementation of prevention protocols relied on overcoming hierarchy and encouraging shared accountability among disciplines [34]. In the Saudi context, these findings may reflect the influence of organizational culture and role expectations; although, such interpretations need to be considered cautiously and viewed as suggestive rather than definitive.

The results highlight ongoing opportunities for hospitals to strengthen the implementation and uptake of urinary catheter nurse-driven protocols in CAUTI prevention. Continued physician resistance and nurse hesitancy impede consistent application. Effective implementation appears contingent upon strong leadership and physician support that facilitate nurse empowerment and confident protocol enactment [2]. Requests from physicians to reinsert catheters may undermine nurses’ confidence, underscoring the importance of clear and structured interprofessional communication to ensure alignment in clinical decisions.

Furthermore, the findings highlight the importance of implementing a nurse-centered approach, as variations in knowledge regarding timely catheter removal, technical competence, and confidence in advocacy may affect clinical practice [35,36] and, if left unaddressed, may hinder full implementation [2]. Effective implementation of nurse-driven urinary catheter removal protocols relies on routine, indication-based daily assessment of catheter necessity, alongside empowering nurses to independently remove unnecessary catheters. These strategies should be supported by targeted education, active physician engagement, and strong leadership endorsement. Enhancing nurses’ competence and fostering professional buy-in are central to embedding nurse-driven urinary catheter removal protocols into routine practice and sustaining effective CAUTI prevention [2].

Our findings regarding knowledge gaps and suboptimal adherence to guidelines are consistent with participants’ experiences, characterized by uncertainty about catheter indications, limited understanding of prevention protocols, and reliance on outdated practices. This is aligned with a study that identified poor awareness toward clinical guidelines, inadequate training, and competing workload demands as major barriers, to adherence to UTI prevention strategies [30]. In the same line, Wu, Bai [22] identified deficiencies in knowledge and the absence of standardized protocols as key barriers to timely catheter removal.

The findings indicate that knowledge gaps influence both nurses’ confidence and their capacity to make timely, evidence-based decisions regarding catheter use. Although education is essential, it is unlikely to be effective in isolation unless supported by clear protocols, strong leadership, audit and feedback mechanisms, and regular access to updated guidance. Research indicates that improving nurses’ knowledge contributes to better UTI and CAUTI outcomes. Educational strategies such as face-to-face instruction, peer learning, tailored training, and structured educational packages have been shown to enhance both prevention knowledge and clinical practice [37,38,39,40,41,42].

The subtheme of inconsistent documentation and lack of standardization further clarifies how unclear records can compromise catheter management. Participants explained that when the reason for catheterization is not documented clearly, nurses become uncertain about whether the catheter should remain in place, especially during shift handovers and busy periods. This finding is consistent with previous work showing that CAUTI prevention depends not only on individual knowledge but also on organizational systems that support access to guidelines, standard assessment, and clear communication [43,44,45,46]. It also aligns with CDC and AHRQ guidance, which emphasize structured documentation and daily review of catheter necessity [24,25,26]. When documentation is inconsistent, continuity of care suffers, interdisciplinary communication becomes weaker, and catheter reassessment is more likely to be delayed. In contrast, standardized documentation improves clarity, supports decision-making, strengthens accountability, and facilitates audit and quality improvement efforts [5,47,48]. Overall, the findings suggest that successful implementation requires alignment across individual, organizational, and process-related factors, rather than isolated interventions.

## 5. Limitations and Strengths of Study

This study has several limitations. The inclusion of a small number of intensive care units from one country may reduce generalizability of the findings to other healthcare settings. While purposive sampling was used, to capture variation in roles and experiences, the sample size remained relatively small, which is considered typical of qualitative studies. Furthermore, the findings rely on self-reported data, which may be affected by recall bias and social desirability effects. Finally, given the study target adult ICU settings, the findings may not be fully applicable to other clinical contexts.

This study has several strengths. The use of a robust qualitative design enabled a detailed exploration of participants’ experiences. The inclusion of perspectives from diverse professional groups assisted with creating a more balanced and comprehensive understanding of the findings, and the application of the CFIR framework provided a strong theoretical foundation to guide interpretation. In addition, the systematic and collaborative approach used data analysis to enhance the rigor of the study, including the credibility and trustworthiness of the results. Overall, the study provides practical and contextually grounded insights that may assist with improving CAUTI prevention practices in ICU settings.

## 6. Implications

Improving CAUTI prevention in Saudi ICUs relies on promoting a supportive unit culture that promotes shared decision-making, regular catheter reassessment, and nurse empowerment. Nurse-driven protocols are more effective when catheter need is routinely reviewed during daily rounds, nurses are confident in initiating removal, and communication across the team is consistent. Nevertheless, challenges such as workload pressures, staffing shortages, and poor documentation highlight the importance of system-level support, including standardized documentation processes, clearly defined responsibilities, and practical tools such as checklists and daily reporting systems.

Protective factors might have a positive impact on nurses’ engagement in infection prevention. For example, an earlier qualitative study indicated that “joy” of helping patients and efficacious coping strategies may minimize stress and burnout. Comparable patterns were evident in this study through nurses’ professional commitment, teamwork, and sense of purpose [49]. These factors may help mitigate the pressures of heavy workloads and organizational challenges, enabling nurses to maintain quality care. Supporting such positive elements by promoting a collaborative culture, recognizing nurses’ contributions, and encouraging reflection may enhance both staff well-being and patient outcomes.

There is a clear need for sustained, targeted educational initiatives aimed at strengthening nurses’ knowledge of catheter use, associated risks, and removal criteria, alongside enhancing confidence in clinical decision-making. Education should extend beyond initial training to include refresher sessions, simulation-based training, case-based learning, and bedside coaching. Furthermore, it should address communication competencies to facilitate assertive participation in interdisciplinary care and mitigate hesitation in catheter removal decisions.

Future studies should prioritize testing practical interventions aimed at overcoming identified barriers, such as reminder systems, standardized documentation tools, and communication strategies. Implementation research and mixed-methods designs are required to evaluate both clinical effectiveness and real-world applicability. In addition, further research should investigate the influence of organizational culture, hierarchical structures, and staffing levels on the adoption and long-term sustainability of nurse-driven protocols across diverse ICU environments.

## 7. Conclusions

This qualitative study provides a context-specific and theoretically informed understanding of the factors influencing the implementation of nurse-driven protocols for catheter-associated urinary tract infection prevention in intensive care units in Saudi Arabia. The findings reveal that implementation is shaped by a complex interplay of determinants across individual, interpersonal, and organizational levels. Key facilitators included effective interprofessional communication, clinical experience, and a strong sense of professional responsibility, while major barriers comprised workload pressures, hierarchical decision-making, knowledge gaps, inconsistent documentation, and cognitive overload, all of which contributed to delayed catheter reassessment and removal.

Overall, the findings indicate that the effectiveness of CAUTI prevention strategies depends not only on the availability of protocols, but also on the presence of supportive systems that enable consistent and confident implementation. Strengthening interprofessional collaboration, enhancing nurse autonomy, standardizing documentation practices, and providing ongoing education and leadership support are critical to improving protocol uptake and sustainability. From an implementation science perspective, these findings highlight the need for multi-level strategies that address both behavioral and structural determinants of practice. Future research should evaluate theory-informed interventions and quantitatively assess the prevalence, patterns, and associated factors of CAUTI prevention practices among nurses to better inform targeted implementation strategies and improve protocol uptake and sustainability.

## Figures and Tables

**Table 1:** Participant Characteristics.

Variable	Category	*n* (%)
Age (years)	<30	7 (30.4)
	30–39	11 (47.8)
	≥40	5 (21.7)
Gender	Female	15 (65.2)
	Male	8 (34.8)
Years of experience	2–5	6 (26.1)
	6–10	9 (39.1)
	11–20	8 (34.8)
Role/Position	ICU nurses	16 (69.6)
	Infection control nurses	4 (17.4)
	Nurse managers	3 (13.0)
Unit/Department	Intensive Care Units (ICUs)	23 (100.0)

**Table 2:** Summary of Themes and Subthemes.

Theme	Subthemes
1. Facilitators of CAUTI Prevention Implementation	1.1 Interprofessional communication and shared decision-making 1.2 Adequate staff experience 1.3 Professional commitment and responsibility
2. Barriers to CAUTI Prevention Implementation	2.1 Heavy workload and staffing pressures 2.2 Physician dominance and nurse hesitation 2.3 Knowledge deficits and poor adherence to guidelines 2.4 Inconsistent documentation and lack of standardization 2.5 Cognitive factors and reduced awareness of catheter presence

## Data Availability

The datasets generated and/or analyzed during the current study are not publicly available due to confidentiality agreements and ethical restrictions but are available from the corresponding author upon reasonable request.

## Supplementary Materials

The following supporting information can be downloaded at: https://www.mdpi.com/article/10.3390/healthcare14121741/s1.
