# Supplementary material for: Exploring Barriers and Facilitators to the Implementation of Nurse-Driven Catheter-Associated Urinary Tract Infection Prevention Protocols in Intensive Care Units in Saudi Arabia: A Qualitative Study
Source: Healthcare (Basel). 2026 Jun 17;14(12):1741. doi: 10.3390/healthcare14121741 (PMC13299888; doi:10.3390/healthcare14121741)
Supplement: Supplementary file 1 [file healthcare-14-01741-s001.zip › healthcare-4319617-supplementary.pdf]

Exploring Barriers and Facilitators to Implementing Nurse-Driven Protocols for CAUTI Prevention in Intensive Care Units in Saudi Arabia: A Qualitative Study

COREQ Checklist

Domain	Item no.	COREQ item	Reported	Manuscript location / notes
Research team and reflexivity	1	Interviewer/facilitator	Yes	Methods 2.3 - Interviews were conducted by the principal investigator.
Research team and reflexivity	2	Credentials	Yes	Methods 2.3 - The principal investigator was trained in qualitative research methods.
Research team and reflexivity	3	Occupation	Partial	Methods 2.3 - The interviewer is identified as the principal investigator; a specific professional role is not detailed.
Research team and reflexivity	4	Gender	Not reported	
Research team and reflexivity	5	Experience and training	Yes	Methods 2.3 - Training in qualitative methods is stated; support from a research team member is also described.
Research team and reflexivity	6	Relationship established before study	Yes	Methods 2.3 / Rigor 2.5 - Rapport was established during interviews.
Research team and reflexivity	7	Participant knowledge of interviewer	Not reported	
Research team and reflexivity	8	Interviewer characteristics/reflexivity	Not reported	
Study design	9	Methodological orientation/theory	Yes	Methods 2 / Methods 2.4 - Qualitative design guided by CFIR and analyzed using thematic analysis.
Study design	10	Sampling strategy	Yes	Methods 2.2 - Purposive sampling with maximum variation.
Study design	11	Method of approach	Yes	Methods 2.2 / 2.6 - Participants were approached after permissions were obtained and invited to participate.
Study design	12	Sample size	Yes	Methods 2.2 / Results 3.1 - Twenty-three participants were recruited.
Study design	13	Non-participation	Not reported	
Study design	14	Setting of data collection	Yes	Methods 2.1 / 2.3 - Adult ICUs in two tertiary hospitals in Riyadh; interviews in private ICU locations.

Study design	15	Presence of non-participants	Not reported	
Study design	16	Sample description	Yes	Methods 2.2 / Results 3.1 - ICU nurses, infection control nurses, and nurse managers; 2-20 years experience.
Study design	17	Interview guide	Yes	Methods 2.3 - A semi-structured interview guide was used.
Study design	18	Repeat interviews	Not reported	
Study design	19	Audio/visual recording	Yes	Methods 2.3 / 2.6 - Interviews were audio-recorded with consent.
Study design	20	Field notes	Not reported	
Study design	21	Interview duration	Yes	Methods 2.3 - Interviews lasted 30-50 minutes.
Study design	22	Data saturation	Yes	Methods 2.2 / 2.3 / Results 3.1 - Recruitment continued until thematic saturation.
Study design	23	Transcripts returned to participants	Not reported	
Analysis and findings	24	Number of data coders	Yes	Methods 2.4 - One author performed primary coding; other authors reviewed transcripts and coding.
Analysis and findings	25	Description of the coding tree	Yes	Methods 2.4 - Codes were organized into a hierarchical tree structure in NVivo.
Analysis and findings	26	Derivation of themes	Yes	Methods 2.4 / Results 3 - Themes were reviewed, refined, defined, and named.
Analysis and findings	27	Software	Yes	Methods 2.4 - NVivo version 8 was used.
Analysis and findings	28	Participant checking	Not reported	
Analysis and findings	29	Quotations presented	Yes	Results 3.2 - Representative quotations are presented for each subtheme.
Analysis and findings	30	Data and findings consistent	Yes	Methods 2.5 / Results 3 / Discussion 4 - Rigor procedures and theme refinement supported consistency.
Analysis and findings	31	Clarity of major themes	Yes	Results 3 / Discussion 4 - Two overarching themes and eight subthemes are clearly reported.
Analysis and findings	32	Clear link between findings and data	Yes	Results 3.2 - Findings are supported by participant quotations and narrative explanation.

Reported status: *Yes = explicitly reported; Partial = partially reported; Not reported = not clearly stated in the manuscript text provided.*
